# Effect of catheter ablation on quality of life in patients with atrial fibrillation and its correlation with arrhythmia outcome

**DOI:** 10.1136/openhrt-2015-000302

**Published:** 2015-09-10

**Authors:** Daniel Raine, Philip Langley, Ewen Shepherd, Stephen Lord, Stephen Murray, Alan Murray, John P Bourke

**Affiliations:** 1 Department of Cardiology, Freeman Hospital, Newcastle upon Tyne, UK; 2 School of Engineering, University of Hull, Hull, UK; 3 Institute of Cellular Medicine, Newcastle University, Newcastle upon Tyne, UK

**Keywords:** ARRHYTHMIAS

## Abstract

**Objective:**

To assess the effect of catheter ablation on atrial fibrillation (AF) symptoms and quality of life (QoL).

**Methods:**

Patients with AF scheduled for ablation were recruited. Pulmonary vein isolation (PVI) was performed and complex fractionated atrial electrogram (CFAE)±linear ablation undertaken in patients in AF despite PVI. QoL and AF symptoms were assessed using SF-36 V2 and Atrial Fibrillation Effect on Quality-of-Life (AFEQT) questionnaires before and 3 months after ablation. Change in QoL scores after ablation was correlated with clinical parameters and the extent of ablation. Magnitude of QoL change was compared between AFEQT and SF-36 physical component summary (PCS) and mental component summary (MCS) scores and correlated with arrhythmia outcome.

**Results:**

80 patients were studied. Summative and individual health scores for both AFEQT (51.5±22.0 vs 81.3±18.2; p<0.01) and SF-36 (PCS 43.3±10.5 vs 47.9±11.3; p<0.01 and MCS 45.0±11.5 vs 51.5±9.4; p<0.01) improved significantly in patients who maintained sinus rhythm after ablation, but not in those with recurrent AF. Improvement in AFEQT (25.4±19) was significantly greater than change in PCS (6.8±6.4; p<0.01) and MCS (8.5±7.9; p<0.01) scores and correlated more closely with arrhythmia outcome (AFEQT r=0.55; PCS r=0.26; MCS r=0.30).

**Conclusions:**

Patients who maintained sinus rhythm after ablation had a significant improvement in AF symptoms and QoL; however, no improvement was observed in patients with recurrent AF. QoL change after ablation did not correlate with baseline clinical parameters or ablation strategy. AF specific QoL scales are more responsive to change and correlate better with ablation outcome.

**Key questions**

**What is already known about this subject?**

- Previous research has shown that catheter ablation improves symptoms and quality of life (QoL) in patients with AF irrespective of arrhythmia outcome and that this improvement is sustained for several years.

**What does this study add?**

- Summative and individual health domain scores for both Atrial Fibrillation Effect on Quality-of-Life (AFEQT) (AF specific) and SF-36 V2 (generic) questionnaires improved significantly in patients who maintained sinus rhythm after ablation; however, there was no improvement in patients with recurrent atrial fibrillation (AF).
- Magnitude of QoL change after ablation did not correlate with clinical parameters (including AF type) or ablation strategy.
- Change in AFEQT was significantly greater than change in SF-36 scores and correlated more closely with arrhythmia outcome following ablation.

**How might this impact on clinical practice?**

- The similar degree of QoL improvement after ablation in paroxysmal and persistent AF supports the continued use of catheter ablation in both patient groups.
- AF specific QoL scales (eg, AFEQT) are more sensitive to change after ablation and correlate better with arrhythmia outcome. Therefore, studies assessing QoL in patients with AF should use AF specific rather than generic QoL scales.

## Introduction

Atrial fibrillation is the most common arrhythmia in clinical practice affecting up to 2% of the general population and is associated with significant morbidity and mortality.^1^ Although some patients with AF are asymptomatic, the majority seek treatment to reduce symptoms and improve quality of life (QoL), which is reduced compared to the general population.^2–4^ Treatment strategies including antiarrhythmic drugs,^5^ ventricular rate control^6^ and catheter ablation^7^ improve QoL particularly if sinus rhythm can be restored and maintained.^8^ However, several studies have reported an improvement in QoL after ablation irrespective of procedural outcome.^9–11^ The most widely validated generic QoL scale is the Medical Outcomes Study Short Form Health Survey (SF-36), which has been successfully used to study a range of cardiovascular conditions including AF.^12^ The greatest weakness of generic QoL measures is that, by design, they reflect general health and functioning, and therefore, results are strongly influenced by patient demographics and comorbidity. Therefore, Spertus *et al*^13^ developed the Atrial Fibrillation Effect on Quality-of-Life (AFEQT) questionnaire as a disease-specific measure to evaluate QoL in AF patients. The *aim* of this study was to assess the effect of catheter ablation on AF symptoms and QoL in patients with paroxysmal or persistent AF using the AFEQT and SF-36 V2 questionnaires.

## Methods

### Patient recruitment

Study participants were recruited from patients scheduled to undergo their first catheter ablation procedure for symptomatic AF. In line with usual practice, class I and III antiarrhythmic drugs were discontinued five half-lives prior to ablation and only restarted in patients with recurrent AF after ablation.

### Ethical approval

This study complies with the Declaration of Helsinki and was granted a favourable ethical opinion by the National Research Ethics North West Committee (REC reference: 11/NW/0476). Written informed consent was obtained from all patients recruited to the study.

### QoL and AF symptom assessment

QoL and AF symptoms were assessed at baseline and 3 months after ablation using the SF-36 V2 and AFEQT questionnaires. Questionnaires were completed without input from study personnel. SF-36 V2 consists of 36 items that assess eight health domains: physical functioning, role limitations because of physical problems, bodily pain, general health perception, vitality, social functioning, role limitations because of emotional problems and mental health. In addition to these eight subscales, physical component summary (PCS) and mental component summary (MCS) scores are also generated, which are normalised to an overall population mean of 50±10.^12^ For all subscales, higher scores represent better functioning and QoL. AFEQT is a 20-item questionnaire that assesses four health domains: symptoms (n=4), daily activities (n=8), treatment concern (n=6) and satisfaction (n=2).^13^ It combines symptoms, functional status and QoL in a single measure and its results have been shown to be reproducible and responsive to change.^13^ Patients’ responses are scored using a seven-point Likert scoring system and a linear relationship is observed between global AFEQT scores and AF severity, with the most severely affected patients having the lowest scores.

### Ablation protocol

#### Pulmonary vein isolation

All patients underwent pulmonary vein isolation (PVI) using one of three ablation technologies: (1) PVAC multielectrode circumferential ablation catheter (Medtronic Ablation Frontiers), (2) Arctic Front Advance 28 mm cryoballoon (Medtronic CryoCath), and (3) Wide-area circumferential ablation guided by the CARTO 3 cardiac mapping system (Biosense Webster). Electrical isolation of the pulmonary veins was confirmed using standard pacing manoeuvres.

#### Left atrial substrate ablation

Additional complex fractionated atrial electrogram (CFAE) and/or linear ablation was performed in patients who remained in AF after PVI, according to the degree of signal complexity and fractionation in the left atrium.

##### CFAE ablation

A detailed analysis of the left atrium and inter-atrial septum was performed to identify CFAEs (focal sites exhibiting constant electrical activity or multicomponent electrograms with cycle length ≤120 ms averaged over a 10 s period), which were then ablated. This process was completed when no residual CFAE sites could be identified or when sinus rhythm was restored by ablation.

##### Linear ablation

A combination of ‘roof line’ (connecting right and left upper pulmonary vein ostia), ‘mitral line’ (connecting left lower pulmonary vein ostium to mitral valve annulus), and ‘inferior line’ (connecting right lower pulmonary vein to coronary sinus) were constructed depending on the degree of signal complexity in each region. Linear ablation was performed until electrograms were no longer recordable or double potentials were evident along the length of each line. Differential pacing manoeuvres were not performed routinely to further confirm conduction block. All patients in AF at the end of the ablation procedure underwent electrical cardioversion.

### Clinical outcome

Clinical outcome was determined by symptom review, 12-lead ECG and 72 h Holter monitoring 3 months after ablation and was divided into two categories: (1) ‘Sinus’ rhythm (no arrhythmia symptoms and no documented AF episodes >30 s); (2) AF recurrence (arrhythmia symptoms and documented AF episodes >30 s).

### Statistical analyses

Continuous variables are expressed as mean±SD. Patient characteristics were compared between paroxysmal and persistent AF groups using Student's independent t test for continuous variables and Pearson's χ^2^ test for categorical variables.

Baseline QoL scores (AFEQT, PCS and MCS) were compared and correlated with clinical parameters using Pearson's correlation. Individual and summative health domains of the AFEQT and SF-36 V2 questionnaires were compared at baseline and 3 months after ablation using Student's paired t tests.

Change in QoL scores after ablation were correlated with clinical parameters using Pearson's correlation (continuous variables) and independent t test (categorical variables) and were compared between different ablation strategies using one-way analysis of variance (ANOVA). Parameters with p<0.05 were entered into a multivariate linear regression model using stepwise selection to assess their independent and combined ability to predict change in QoL after ablation. After multivariate analysis, parameters with p<0.10 were retained. Standardised coefficients (β) and corresponding p values are reported. The magnitude of QoL change after ablation was compared between AFEQT, PCS and MCS scores using one-way ANOVA and correlation coefficients for the three QoL scores and ablation outcome were compared using Fisher r-to-z transformation. All tests were two-tailed and p<0.05 was considered statistically significant.

## Results

The clinical characteristics of the 80 consecutive patients recruited to the study are shown in table 1. The mean age was 57±10 years and 73% were male. The mean duration of AF history was 4±3 years. Patients with persistent AF (n=36) had a significantly shorter AF history, larger left atrial volume and lower left ventricular ejection fraction than those with paroxysmal AF (n=44). There were no procedural complications and 54/80 (68%) patients (77% paroxysmal; 56% persistent AF) maintained sinus rhythm 3 months after ablation.

**Table 1:** Patient characteristics

	Paroxysmal AF (n=44)	Persistent AF (n=36)	p Value
Age (years)	56.9±10.5	58.2±9.5	0.58
Male gender	30 (68%)	28 (78%)	0.45
AF history (years)	4.4±3.4	2.9±2.7	0.04
LA volume (mL)	53.1±17.2	64.6±20.1	<0.01
LVEF (%)	54.4±2.2	50.9±7.8	0.02
Hypertension	12 (27%)	14 (39%)	0.34
Diabetes	2 (5%)	5 (14%)	0.23
Smoking	22 (50%)	17 (47%)	0.83

AF, atrial fibrillation; LA, left atrial; LVEF, left ventricular ejection fraction.

### Effect of ablation on QoL

**Figure 1:**
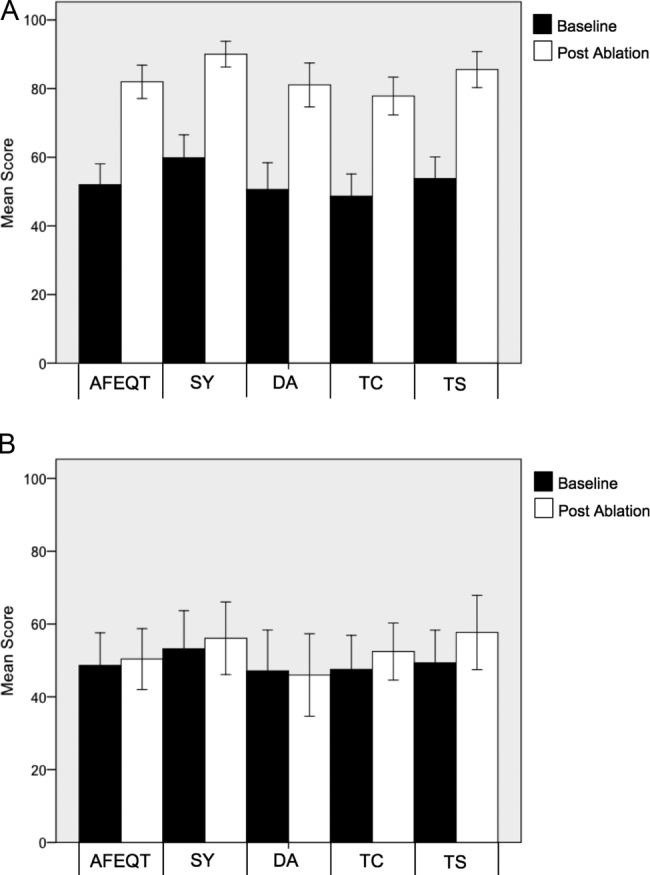
Mean individual and summative AFEQT scores before and 3 months after ablation. (A) Patients who maintained sinus rhythm post ablation; (B) Patients with recurrent AF. AFEQT, Atrial Fibrillation Effect on Quality-of-Life; DA, daily activities; SY, symptoms; TC, treatment concern; TS, treatment satisfaction.

**Figure 2:**
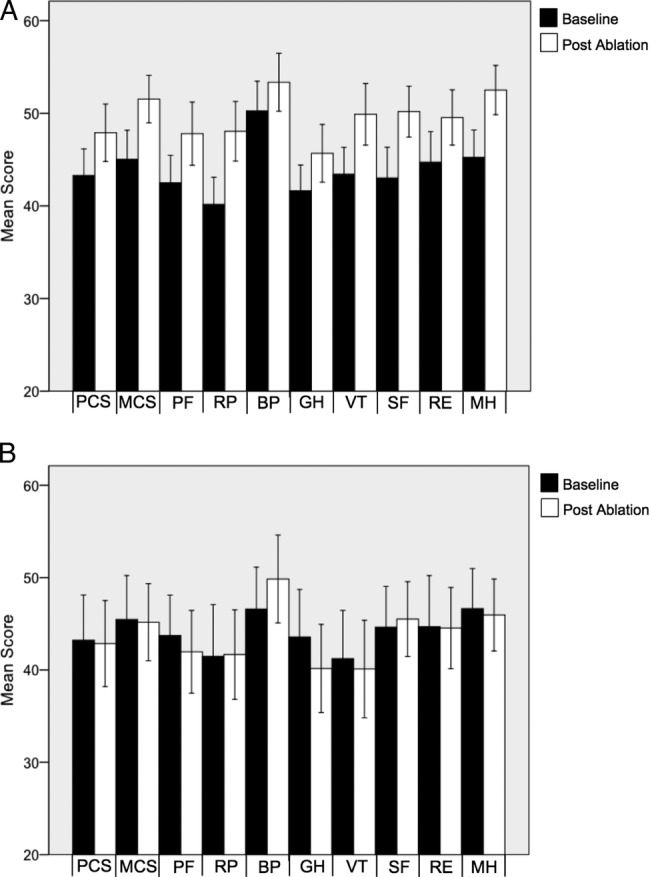
Mean individual and summative SF-36 V2 scores before and 3 months after ablation. (A) Patients who maintained sinus rhythm post ablation; (B) Patients with recurrent AF. PCS, physical component summary; MCS, mental component summary; PF, physical functioning; RP, role limitations because of physical problems; BP, bodily pain; GH, general health perception; VT, vitality; SF, social functioning; RE, role limitations because of emotional problems; MH, mental health.

There was a significant increase in the summative and individual health domain scores for both AFEQT (51.5±22.0 vs 81.3±18.2; p<0.01) and SF-36 V2 (PCS 43.3±10.5 vs 47.9±11.3; p<0.01 and MCS 45.0±11.5 vs 51.5±9.4; p<0.01) questionnaires in patients who maintained sinus rhythm 3 months after ablation (figures 1A and 2A).

However, there was no significant change in summative or individual scores from either questionnaire in patients with recurrent AF (figures 1B and 2B). The magnitude of change in AFEQT score (25.4±19) after ablation was significantly greater than the change in PCS (6.8±6.4; p<0.01) or MCS (8.5±7.9; p<0.01) scores and there was no significant difference observed between the PCS and MCS scores (p=0.14). Change in AFEQT score after ablation correlated more closely with 3-month outcome (r=0.55) than PCS (r=0.26; p=0.03) or MCS (r=0.30; p=0.05) scores.

In univariate analysis, there was no significant correlation between clinical parameters and change in QoL scores after ablation (table 2). Of note, there was no difference in the magnitude of change in QoL after ablation between paroxysmal and persistent AF groups (AFEQT 24.3±24.3 vs 16.3±23.4, p=0.14; PCS 3.0±9.2 vs 3.1±8.7, p=0.98; MCS 5.8±11.2 vs 2.6±10.1, p=0.19). However, higher QoL scores at baseline and AF recurrence correlated with smaller changes in QoL scores after ablation for both questionnaires. In multivariate analysis, higher AFEQT, PCS and MCS scores preablation and AF recurrence were independent predictors of a smaller change in their respective QoL scores after ablation (table 3).

**Table 2:** Change in QoL scores after ablation: relationship with clinical parameters and ablation outcome

	AFEQT *R/T (P)*	PCS *R/T (P)*	MCS *R/T (P)*
Age	−0.02 (0.87)	0.05 (0.70)	−0.16 (0.15)
Female gender	0.28 (0.78)	−1.14 (0.26)	1.31 (0.19)
Persistent AF	1.48 (0.14)	−0.03 (0.98)	1.33 (0.19)
AF history	−0.09 (0.44)	−0.18 (0.11)	−0.01 (0.96)
LA volume	<−0.01 (0.98)	0.06 (0.63)	−0.34 (<0.01)
LVEF	0.06 (0.61)	−0.05 (0.68)	0.10 (0.38)
Hypertension	−0.57 (0.57)	−0.01 (0.99)	−0.49 (0.63)
Diabetes	1.31 (0.20)	0.95 (0.34)	−0.23 (0.82)
Smoking	−1.96 (0.05)	−0.40 (0.69)	−0.67 (0.51)
Baseline			
AFEQT	−**0.47 (<0.01)**	−0.10 (0.37)	−0.13 (0.25)
PCS	−**0.22 (0.05)**	−**0.34 (<0.01)**	0.20 (0.08)
MCS	−0.12 (0.30)	0.16 (0.17)	−0.54 (<0.01)
Three-month outcome	−**5.79 (<0.01)**	−**2.39 (0.02)**	−**2.72 (<0.01)**

AF, atrial fibrillation; AFEQT, Atrial Fibrillation Effect on Quality-of-Life; LA, left atrial; LVEF, left ventricular ejection fraction; MCS, mental component summary; PCS, physical component summary.

R values from Pearson's correlation (continuous variables) and T values from independent t tests (categorical variables) are shown.

**Table 3:** Multivariate predictors of change in QoL scores after ablation

Parameter	Standardised coefficient (β)	p Value
AFEQT		
AF recurrence	0.57	<0.01
Baseline AFEQT	−0.56	<0.01
PCS		
Baseline PCS	−0.40	<0.01
Baseline MCS	0.25	0.02
AF recurrence	0.23	0.03
MCS		
Baseline MCS	−0.68	<0.01
AF recurrence	0.28	<0.01
Baseline PCS	0.27	<0.01

AF, atrial fibrillation; AFEQT, Atrial Fibrillation Effect on Quality-of-Life; MCS, mental component summary; PCS, physical component summary.

### Change in QoL according to ablation strategy

AFEQT scores increased significantly after PVI, PVI+Linear and PVI+Linear+CFAE ablation indicating an improvement in QoL (table 4). AFEQT scores also increased after PVI+CFAE ablation although this did not meet statistical significance (p=0.12). There was a significant increase in PCS and MCS scores after PVI and an increase in MCS score after PVI+Linear ablation. However, there was no significant change in PCS and MCS scores after the other ablation strategies. Of note, there was no significant difference in the magnitude of change in QoL scores between the four ablation strategies (AFEQT p=0.67; PCS p=0.49; MCS p=0.29).

**Table 4:** Change in QoL scores according to ablation strategy

	AFEQT			PCS			MCS		
	Baseline	3 months	p Value	Baseline	3 months	p Value	Baseline	3 months	p Value
PVI (n=45)	53.4±22.3	76.7±20.9	<0.01	43.8±10.7	47.0±11.0	0.02	44.3±12.1	49.8±9.1	<0.01
PVI+Linear (n=17)	47.6±23.0	62.7±25.5	0.04	44.5±9.8	44.9±10.4	0.82	45.4±10.5	50.9±9.4	0.04
PVI+CFAE (n=8)	39.2±15.3	56.9±22.3	0.12	38.2±12.5	43.2±11.7	0.26	44.1±13.8	47.0±11.6	0.46
PVI+Linear+CFAE (n=10)	52.1±23.2	72.5±29.0	0.02	43.0±13.0	48.2±15.8	0.14	49.5±8.3	48.0±14.1	0.65

CFAE, complex fractionated atrial electrogram; MCS, mental component summary; PCS, physical component summary; PVI, pulmonary vein isolation; QoL, quality of life.

### Relationship between clinical variables and QoL

Baseline AFEQT scores correlated closely with PCS scores (r=0.64, p<0.01) and moderately with MCS scores (r=0.43, p<0.01). However, there was no correlation between PCS and MCS scores (r=0.16, p=0.15). Hypertension was associated with a lower baseline AFEQT score with a trend towards a lower MCS score (table 5). Older age and persistent AF were associated with a lower PCS score. Diabetes was associated with a lower MCS score with a trend towards a lower PCS score. Larger left atrial volume was associated with a higher MCS score, which is unlikely to be clinically meaningful.

**Table 5:** Baseline QoL scores and clinical parameters

	AFEQT *R/T (P)*	PCS *R/T (P)*	MCS *R/T (P)*
Age	−0.14 (0.22)	−0.35 (<0.01)	0.05 (0.69)
Female gender	1.01 (0.31)	1.13 (0.26)	−0.14 (0.89)
Persistent AF	1.64 (0.10)	2.05 (0.04)	0 (1)
AF history	0.14 (0.22)	0.20 (0.09)	−0.04 (0.76)
LA volume	−0.03 (0.82)	−0.20 (0.09)	0.33 (<0.01)
LVEF	−0.01 (0.96)	0.12 (0.29)	−0.08 (0.47)
Hypertension	2.22 (0.03)	1.43 (0.16)	1.93 (0.06)
Diabetes	1.38 (0.17)	1.81 (0.07)	2.64 (0.01)
Smoking	1.21 (0.23)	0.35 (0.73)	0.96 (0.34)

R values from Pearson's correlation (continuous variables) and T values from independent t tests (categorical variables) are shown.

AFEQT, Atrial Fibrillation Effect on Quality-of-Life; AF, atrial fibrillation; CFAE, complex fractionated atrial electrogram; LA, left atrial; LVEF, left ventricular ejection fraction; MCS, mental component summary; PCS, physical component summary; PVI, pulmonary vein isolation; QoL, quality of life.

## Discussion

### Effect of ablation on QoL

The main finding of this prospective study is the significant improvement in AF symptoms and QoL in patients who maintained sinus rhythm 3 months after ablation and the contrasting lack of improvement in patients with recurrent AF. This finding was consistent across all individual and summative components of the AFEQT and SF-36 V2 questionnaires. The mean increase in AFEQT score of 30 points in patients who maintained sinus rhythm is consistent with a marked improvement in QoL.^14^ The expected lack of QoL improvement in patients with recurrent AF after ablation is clinically coherent and supported by Fiala *et al*^15^ and Mohanty *et al*.^16^ However, other groups have reported significant QoL improvements regardless of arrhythmia outcome.^9–11^ This might be explained by a transition from symptomatic to asymptomatic AF, reduction in AF burden short of AF abolition or placebo effect.

Previous studies have shown that AF specific QoL scales are more responsive to QoL change in this population and correlate better with ablation outcome.^9^ ^13^ This is corroborated by the greater change in QoL score after ablation and the stronger correlation with ablation outcome observed with the AFEQT questionnaire in this study. There was no significant correlation between the clinical parameters examined (including AF type) and change in QoL scores after ablation, as shown by others.^9–11^

However, Bulkova *et al*^17^ reported that long-standing persistent AF, younger age and a shorter history of AF were associated with an improvement in QoL 3 years after ablation. The different patient populations and longer duration of follow-up could explain the discordance in results. In agreement with others, higher QoL scores pre-ablation and AF recurrence were independent predictors of a smaller change in the respective QoL score after ablation.^9^ ^11^ ^14^ This suggests a ceiling effect, as there is less potential for improvement after ablation in those with preserved QoL beforehand.

### Change in QoL according to ablation strategy

Mantovan *et al*^11^ reported a significant improvement in PCS and MCS scores after PVI, CFAE and PVI+CFAE ablation strategies with the exception of MCS in the CFAE ablation group. In our study, we observed a significant increase in all three QoL scores after PVI; however, there was no significant improvement in PCS and MCS scores after PVI+CFAE ablation. This is likely a chance effect on account of the small number of patients in this subgroup. To our knowledge, the effect of PVI+Linear ablation on QoL has not been previously reported. In this study, PVI+Linear ablation was associated with a significant improvement in AFEQT and MCS scores; however, there was no significant difference in PCS scores. Of note, there was no significant difference in the magnitude of QoL change after ablation between the four ablation strategies.

### Relationship between clinical variables and QoL

This study showed no correlation between gender and QoL, in contrast to Reynolds *et al*^18^ who reported that female patients were more symptomatic from AF with a consequent reduction in QoL. In addition, they reported that older patients (>65 years) had fewer AF symptoms than their younger counterparts, which was not observed in this study. The disparity in results may be explained by different patient populations and eras of AF management as they studied patients with new-onset AF who were managed pharmacologically prior to the development of AF ablation; whereas, we studied patients referred for ablation who, by their very nature, have a high AF symptom burden and reduced QoL.

Hypertension was associated with a higher symptom burden and reduced QoL secondary to AF in this study, which has not been previously reported. Persistent AF was associated with lower physical health scores than paroxysmal AF, as shown by Bulkova *et al*.^17^ However, there was no difference in AF symptom burden between the two groups, which contradicts the common belief that symptoms regress as AF progresses from paroxysmal to persistent forms. Finally, diabetes was associated with lower mental health and a trend towards lower physical health scores in this study, which supports its negative impact on QoL.

### Limitations

First, the choice of ablation strategy after PVI was not randomised and was dependent on the operator and the degree of signal complexity in the left atrium. Second, this was a prospective cohort study without a randomised control group comparing AF ablation to best non-ablative management (rate vs rhythm control). However, the superiority of ablation over antiarrhythmic drugs in improving QoL has been demonstrated consistently. Third, AF recurrence was determined by patients’ symptoms, 12-lead ECG and 72 h Holter monitoring 3 months after ablation and not by continuous ECG monitoring. Although, we cannot completely exclude asymptomatic AF episodes in the ‘sinus rhythm’ group, we consider this unlikely given previous AF symptoms. Fourth, change in QoL was assessed 3 months after ablation in line with the first outpatient clinic review and therefore, the longer-term effect of ablation on QoL is unknown. Finally, patients enrolled in this study had been referred for catheter ablation and therefore, represent a select cohort with a high AF symptom burden. Extrapolation of the benefits demonstrated to a wider population requires caution.

### Conclusions

Patients who maintained sinus rhythm after ablation had a significant improvement in AF symptoms and QoL. No improvement was observed in patients with recurrent AF. QoL did not correlate with either baseline clinical parameters or extent of ablation.

AF specific QoL scales are more responsive to change than generic measures and correlate better with arrhythmia outcome.
